# Erythrocyte Efferocytosis by the Arterial Wall Promotes Oxidation in Early-Stage Atheroma in Humans

**DOI:** 10.3389/fcvm.2017.00043

**Published:** 2017-08-02

**Authors:** Sandrine Delbosc, Richard Graham Bayles, Jamila Laschet, Veronique Ollivier, Benoit Ho-Tin-Noé, Ziad Touat, Catherine Deschildre, Marion Morvan, Liliane Louedec, Laurent Gouya, Kevin Guedj, Antonino Nicoletti, Jean-Baptiste Michel

**Affiliations:** ^1^UMRS 1148, INSERM, Paris 7-Denis Diderot University, Hôpital Xavier Bichat, Paris, France; ^2^Département Hospitalo-Universitaire DHU “FIRE”, Paris, France; ^3^UMRS 1149, INSERM, Paris 7-Denis Diderot University, Hôpital Xavier Bichat, Paris, France

**Keywords:** smooth muscle cells, oxidative stress, iron, fatty streaks, hemoglobin, translational studies, vascular biology, atherosclerosis

## Abstract

**Background:**

Since red blood cells (RBCs) are the predominant cellular blood component interacting with the arterial wall, we explored the role of RBCs efferocytosis by vascular smooth muscle cells (vSMCs) in the initiation of human atheroma.

**Methods and results:**

The comparison of human healthy aortas with aortic fatty streaks or fibroatheromas revealed that RBC angiophagy is implicated from the earliest stages of atherogenesis, as documented by the concomitant detection of redox-active iron, hemoglobin, glycophorin A, and ceroids. RBCs infiltration in the arterial wall was associated with local lipid and protein oxidation, as well as vascular response (expression of heme oxygenase-1 and of genes related to iron metabolism as well as those encoding for phagocytosis). These effects were recapitulated *in vitro* when vSMCs were co-cultured with phosphatidyl-exposing senescent (s) RBCs but not with fresh RBCs. VSMCs engulfing sRBC increased their intracellular iron content, accumulated hemoglobin, lipids, and activated their phagolysosomes. Strikingly, injections of sRBCs into rats promoted iron accumulation in the aortic wall. In rabbits, hypercholesterolemia increased circulating senescent RBCs and induced the subendothelial accumulation of iron-rich phagocytic foam cells. RBCs bring cholesterol and iron/heme into the vascular wall and interact with vSMCs that phagocytize them.

**Conclusion:**

This study presents a previously unforeseen mechanism of plaque formation that implicates intimal RBC infiltration as one of the initial triggers for foam cell formation and intimal oxidation. Pathogenic effects exerted by several metabolic and hemodynamic factors may rely on their effect on RBC biology, thereby impacting how RBCs interact with the vascular wall.

## Introduction

Atherothrombosis is a consequence of multiple interactions between blood circulating components and the arterial wall. These initial interactions involve both radial convection and intimal retention of low-density lipoprotein (LDL) (1), and collision of blood cells [platelet, red blood cell (RBC), and leukocytes] with the wall (2). Such interactions are greatly potentiated by local hemorheology modified by non-uniform geometries of the endoluminal wall surface (3–5), which could explain the focal nature of the disease. Intimal oxidative processes characterize early human and rabbit atheroma (6), but the triggers of these remain uncertain and can result in site-specific injuries of the intima caused by circulating blood cells.

In previous and recent human (7–9) and murine (10–12) studies, intimal vascular smooth muscle cells (vSMCs) were identified as important contributors to the foam cells and phagocyte contingents to the atherosclerotic wall. vSMCs from the tunica media migrate into the intima and respond to initial lipid and cellular injuries by engaging their clonality (12) and phenotypic plasticity (13). The roles of leukocytes (14) and platelets (15) in the initiation of atherothrombosis have been extensively investigated. Despite their high predominance as circulating cells, the roles of RBCs and of their hemoglobin cargo have been less explored (16). Nevertheless, it has been demonstrated that free heme by increasing granulocyte ROS production (17) or LDL oxidation (18) participated to the endothelial injury. This point has also been confirmed with free hemoglobin cytotoxicity for the arterial wall (19). However, the direct role of RBC collision and penetration within the arterial wall and its impact on resident vSMCs behind the endothelium have not yet described in human atherosclerotic tissue.

Key studies reported by the groups of Arbustini et al. (20) and Kolodgie (21) have demonstrated that intraparietal RBCs, through intraplaque hemorrhages and hematoma, can also critically contribute to lipid core expansion in relation to their membrane cholesterol content. These observations were then expanded to oxidation (22). Due to their phenotypic plasticity, human vSMCs possess both endocytic (8) and efferocytic (23) capacities, which are associated with atheroma development. vSMCs have been reported to transform into foam cells when they internalize macromolecules, such as aggregated or oxidized LDL (24), or microparticles (25). Importantly, we have reported that this is also the case following the engulfment and intracellular metabolism of senescent RBCs (sRBCs) (23).

In the present study, we studied how the interactions between RBCs, and the wall intima could impact the early stages of human atheroma. To accomplish this, we evaluated intraparietal iron and hemoglobin content and tissue-associated oxidative stress in human fatty streaks (FSs) and fibro-atheroma. Iron retention was found to be linked to the metabolism of RBC components predominantly achieved by vSMCs. We tested the ability of cultured human vSMCs to perform erythrophagy *in vitro*, and analyzed its consequences on lipophagy, iron retention, and oxidation. We further confirmed this relationship *in vivo* using the foam cell-rich hypercholesterolemic rabbit model, and a rat model created by subjecting animals to repeated intravenous injections of sRBCs.

## Materials and Methods

### Tissue Sampling

Human aortas were consecutively collected from deceased organ donors from 2010 to 2013 under the authorization of the French Biomedicine Agency (PFS 09-007). After macroscopic examination, the aortas were classified according to Stary classification (26) and the Virmani word list (27) into three groups: healthy aortas (HAs, *n* = 34), aortas with FS (*n* = 67), and aortas with fibrolipidic initial plaques [fibroatheromas (FAs), *n* = 56]. A small portion of tissue from each sample was fixed in 3.7% paraformaldehyde for classical histology and immunochemistry assessments. For the HA samples, it was practically and virtually impossible to separate and independently process the tunica intima. For these samples, the adventitia was carefully removed, and only the results obtained from the tunica media are presented. For the FS and FA samples, the adventitia was also carefully removed, and the dissected intimal lesions and media layers were processed separately. Tunica medias from HA, FS, and FA, as well as intimal lesions from FS and FA were either incubated in RPMI-1640 culture medium (24 h, 37°C) to obtain tissue-conditioned media or placed in RNALater (24 h, 4°C) for RNA extraction. The isolated tunica intimas (FS *n* = 8, FA *n* = 8) were directly fixed in paraformaldehyde for subsequent “whole mount” immunohistochemistry.

### Human Vascular SMC Culture

Vascular smooth muscle cells were isolated from human HAs. As previously described (28), the tunica media was separated from the tunica adventitia before being cut into small pieces and digested in collagenase/elastase. Cells were subcultured to the sixth passage prior to experimentation. Purity of the culture was assessed by immunostaining of SMC α-actin and transgelin (SM22-α) (data not shown).

### Experimental Models for RBC-Triggered Arterial Wall Injuries

#### Rabbit Model

Five New Zealand white rabbits were fed a high-fat diet (4.7% coconut oil and 0.3% cholesterol-enriched diet, Research Diet) for 6 weeks as previously described (29, 30). The rabbits were sacrificed under deep anesthesia (50 mg/kg, IP), and their aortas were removed and fixed in 3.7% paraformaldehyde.

#### Rat Model

Thirty-eight inbred male Lewis rats were treated with two intravenous injections (2 × 10^8^ cells in 500 µl, 2 and 4 days after surgery) of sRBCs (see below) isolated from syngeneic Lewis rats. Controls (*n* = 38) were injected with the same volume of saline. Prior to RBC administration, 20 rats in each of these groups underwent a balloon injury of the infrarenal abdominal aorta or a sham operation (*n* = 18). In each of these subsets, the rats either underwent splenectomy (*n* = 10) or did not (*n* = 8). The surgical procedure was as follows. After intraperitoneal administration of pentobarbital anesthesia, a median laparotomy was performed, and the left iliac artery was isolated and ligated. The aorta was then isolated and clamped below the renal arteries. A 3F Fogarty balloon catheter was inserted by the right iliac artery, pushed upwards into the aorta, and inflated. The inflated balloon was gently removed three times, causing injury to the abdominal aorta, as previously described (31). Splenectomy was performed by ligature of the spleen vascular pedicles. 10 days after surgery, the rats were anesthetized with sodium pentobarbital (50 mg/kg, IP) and sacrificed. Their aortas were removed and either fixed in 3.7% paraformaldehyde or snap-frozen for iron content determination.

All experimental and animal care procedures complied with the principles formulated by the National Society for Medical Research (animal facility agreement: No. B75-18-03). This study was performed under the advice of our institution’s animal ethical committee (authorization no. 75-101) and complied with all guidelines for animal experimentation set forth by the French Ministry of Agriculture.

### Senescent RBCs

Peripheral blood was collected from healthy volunteers and Lewis rats in heparinized tubes. Following this, the blood was centrifuged at 120 *g* for 15 min, and the plasma and the buffy coat were removed. The pellet containing RBCs was then mixed (1:1) with a dextran solution (2% in HBSS) for 15 min, centrifuged (1,500 *g* for 5 min), and washed four times with HBSS (with Ca^2+^ and Mg^2+^) supplemented with 1% antibiotic. Finally, to obtain sRBCs, the pellet was resuspended in HBSS supplemented with 0.1% glucose and incubated at 37°C for 5 days. RBCs incubated in HBSS without glucose (fRBCs) were used as controls. RBC senescence was validated by evaluating phosphatidylserine (PS; annexin V-FITC binding) exposure on cell membranes by flow cytometry. RBCs were washed with PBS before *in vivo* (rats) or *in vitro* (cell biology) use in order to limit the direct effect of free hemoglobin.

### Tissue Hemoglobin Content Oxidative Stress Markers, Glycophorin A, and Iron Content

Hemoglobin content was estimated by the formic acid method. The acid induced CO (carbon monoxide) formation which reacted with hemoglobin to create a reversible carboxy-hemoglobin. 30 µL of the samples or the standards and 70 µL of formic acid were added to 96-well plates. Absorbance was read immediately at 420 nm. Hemoglobin values were calculated based on the standard curve (free hemoglobin).

Thiobarbituric acid reactive substance (TBARS), AOPPs (markers of oxidized lipids and proteins, respectively) (28), and Gly-A concentrations (ELISA kit from USCNK) were determined in the conditioned medium of human atherosclerotic lesions. To determine iron content, tissue samples were incubated for 48 h in an extraction solution containing 6.25 mL H_2_O, 1.25 g trichloroacetic acid, 3.225 mL 36% HCl, and 0.375 mL 98% thioglycolic acid. The samples were then diluted 1:10 in 1.5 M sodium acetate. Iron concentrations were determined based on spectrophotometric readings taken at 535 nm and expressed as mM/mg of tissue.

### Incubation of RBCs and Hb with vSMCs and ROS Production

Intracellular ROS production by vSMCs was determined using 5- and 6-chloromethyl-2,7′-dichlorodihydrofluorescein diacetate, acetyl ester (CM-H2DCFDA, Life Technologies) (28). vSMCs were seeded in a 96-well plate and starved overnight after reaching 80% confluence. Senescent or fresh RBCs (1 × 10^6^ cells) were added to the vSMCs and incubated for 3 days. In separate experiments, vSMCs were incubated with increasing concentrations of Hb (0–100 µM), in the presence or the absence of desferoxiamine (20 µM). After washing the vSMCs, 5 µM CM-H2DCFDA was added to each well, and fluorescence intensity was monitored every 2 min for 2 h. The results are expressed as oxidation rates (fluorescence intensity/min; mean ± SEM).

### Phagocytosis Assay

Vascular smooth muscle cells were seeded and grown in a 6-well plate until 80% confluent. Fresh or senescent RBCs (1 × 10^6^ cells) were labeled with CFSE according to the manufacturer’s instructions and incubated with the vSMCs for 3 days. At the end of the experiment, the supernatant was removed, and the cells were washed three times in PBS and dissociated using trypsin. After being centrifuged (600 × *g*, 5 min), the cells were resuspended in 4% PFA and analyzed at 40× magnification on an ImageStream flow cytometer. In brief, images of vSMCs that had been cultured in the presence of fresh or senescent RBCs were used for measuring the internalization coefficient (internalization function of the IDEAS^®^ analysis software from Amnis). The software calculates a ratio based on the fluorescence intensity inside the cell compared with the fluorescence intensity of the entire cell. To discriminate both signals, we defined a mask smaller than the cell in which internalization was measured. An elevated ratio indicates a high internalization coefficient.

### Tissue and Cell RT-PCR

Aortic tunica intima and media tunicas were independently cryo-pulverized in liquid nitrogen using a freezer mill (model 6750 SPEX SamplePrep). Total RNA was extracted from the pulverized tissues and vSMCs using TRIzol reagent (Life Technologies) according to the manufacturer’s protocol. Reverse transcription was performed using the MMLV enzyme (Life Technologies), and real-time PCR was conducted in a LightCycler system using a SYBR green detection kit (Roche Applied Science) with specific primers (Table 1).

**Table 1:** Primers used for real-time PCR.

	Forward	Reverse
HO-1	5′ AACTTTCAGAAGGGCCAGGT 3′	5′ TGTTGCGCTCAATCTCCTC 3′
NRAMP-1	5′ ATGACAGGTGACAAGGGTCC3′	5′ GGGCTGGAGATGGAACCATAG 3′
Ferritin	5′ CTGGAGCTCTACGCCTCCTA 3′	5′ CCACATCATCGCGGTCAAAG 3′
TIM-3	5′ TCCAAGGATGCTTACCACCAG 3′	5′ GCCAATGTGGATATTTGTGTTAGATT 3′
STAB1	5′ TCAAGTCGCTGCCTGCATAG 3′	5′ CAGCGTGCCAAAGAAACCAG 3′
MR	5′ GACCAAAAGGCCAGAAAGGG 3′	5′ CAAGCTCCTACAGACGACCT 3′
HPRT	5′ TGAGGATTTGGAAAGGGTGT 3′	5′ GAGCACACAGAGGGCTACAA 3′

### Histology and Immunohistochemistry

Aortic tissues were embedded in paraffin and cut into 5-μm-thick sections. For their morphological characterization, we used hematoxylin and eosin and Masson’s trichrome staining. DiAminoBenzidin (DAB) staining was used to determine the presence of peroxidase activity (32). Ferric iron (FeIII) storage (present mostly in association with ferritin) was visualized by the ferrocyanide precipitation (Perl’s Prussian blue staining). Nevertheless, Prussian blue precipitation is highly specific for FeIII storage, but poorly sensitive to detect other forms of redox-active iron. Therefore, the presence of the low-molecular-weight iron pool (33) and of highly redox-active Fe (34) was assessed by the combination of Perls and DAB staining, successively associating two redox pairs, Fe^+++^/Fe^++^ (Perls) and H_2_O_2_/H_2_O leading to DAB polymerization by oxidation. In these reactions, endogenous oxidases were not primarily blocked, since redox-active iron pair catalyze endogenous oxidase reactions (Haber–Weiss and Fenton reactions) detected by DAB polymerization (32). Negative controls are DAB alone and Perls alone.

To detect the presence of fluorescent ceroids (λ excitation 510–560 nm, λ emission 590 nm), fixed sections were observed using a Zeiss Axiovert 200 M inverted microscope equipped with an AxioCam MRm version 3 camera, an ApoTome^®^ system and AxioVision^®^ image capture software. In addition, positive Oil Red O (ORO) staining indicated ceroid ability to retain lipids [persistent staining despite delipidation during paraffin removal by detergents (35)]. Fluorescence measurements taken at 330–380 nm (U.V.) were used to compare sections with or without ORO staining of ceroids. Gly-A (Dako) immunostaining was used to visualize RBC membranes on paraffin sections of early atheroma lesions. Immunostaining of smooth muscle cell-specific myosin (MYH-11) was performed using the monoclonal antibody, ID8 (EMD, Millipore). The immunodetection of hemoglobin was performed using a rabbit polyclonal antibody (H4890, Sigma).

Because no antibody that recognizes rabbit Gly-A is currently available, we used *Bandeiraea simplicifolia* isolectin B4 (GSL-B4, L2140, 10 μg/ml Sigma Chemical), a previously described ligand of rabbit RBC membrane glycoconjugates bearing α-d-galactose (30). Rabbit phagocytes were detected using a mouse monoclonal anti-rabbit RAM11 antibody [M063, dilution 1:500, Dako North America Inc. (29)]. These immunostaining experiments were performed on paraformaldehyde-fixed rabbit aorta cross-sections. A horseradish peroxidase-conjugated secondary antibody and DAB were used to reveal the primary antibody.

### Statistics

All the results are expressed as the mean ± SEM of multiple experiments. Statistical analyses were performed using Prism 5 (GraphPad software). Differences in event frequencies were assessed using chi-squared tests. Differences between two groups were assessed with Mann–Whitney tests and were considered significant at *p* < 0.05.

## Results

### Iron and Hemoglobin Deposits Are Present in Early Atherosclerotic Human Aortas

We first evaluated the presence of iron deposits in consecutively collected human FSs and FAs or in human aortas without atherosclerotic lesions (HAs). The average age of the patients with FAs was significantly higher (*p* < 0.001) than that with HAs. The male gender was predominant regardless of aorta status (*p* < 0.001). HAs were more frequent in the thoracic tract than in the abdominal tract (*p* < 0.001, Table 2).

**Table 2:** Stages of early lesions in the thoracic and abdominal aortas according to subject age and gender.

	HA (*n* = 34)	FS (*n* = 67)	FA (*n* = 56)
Age (years)	48 ± 17	53 ± 17	63 ± 12**
Gender (% male)	69%	59%	68%
Thoracic aorta	62%***	14%	24%
Abdominal aorta	32%	25%	43%
Perls’ + DiAminoBenzidin (DAB)	22%***	84%	90%
DAB (alone)	0%	16%	26%
Perls’ (alone)	0%	9%	39%
Ceroid detection	23%***	71%	94%

*The frequencies with which iron and ceroids were detected are listed at the bottom of the table*.

**p < 0.01, ***p < 0.0001, chi-squared test.

**Figure 1:**
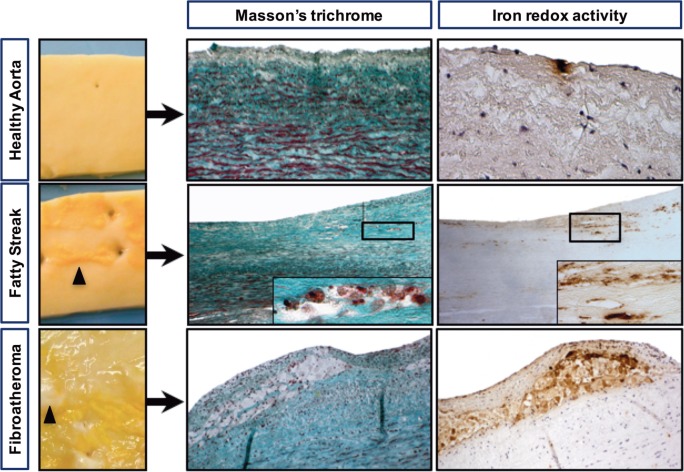
Iron deposits are frequently detected in early-stage atheroma lesions. Macroscopic pictures of a healthy aorta (smooth luminal interface), a fatty streak with subendothelial yellow deposits (black arrowheads) and a fibroatheroma with yellow deposits covered with a white capsule, forming initial plaques (black arrowheads). Visualization of foam cells was accomplished by Masson’s trichrome staining, and redox-active iron was visualized by Perls reaction followed by DiAminoBenzidin (DAB) polymerization (see materials and methods). Perls staining alone and DAB alone were strictly negative (not shown).

**Figure 2:**
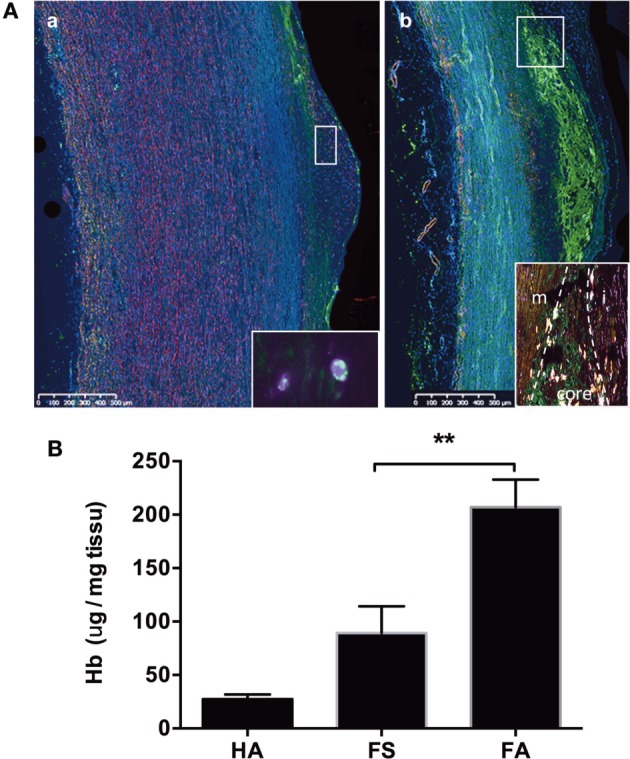
Hemoglobin deposits in fatty streaks and fibro-atheroma lesions. Detection of Hb (green) and SMC myosin (pink) [**(A)**, a] in the endothelium and subendothelial space of a fatty streak [inset: high magnification (×100) showing the intracellular co-localization of myosin and Hb (merged yellow)], and [**(A)**, b] in in the lipid core (between the fibrocellular cap and the media) of a fibroatheroma lesion [inset: high magnification of the plaque shoulder (m, media; i, intima; core shoulder) showing the presence of SMC myosin (pink) and hemoglobin (green), and their frequent localization (merged yellow)]. For negative control, there is no green autofluorescence in the lipid core (not shown). **(B)** Measurement of heme content in FS and FA tissue by formic acid reaction. **p < 0.01; Mann–Whitney’s test.

As depicted in Figure 1, HAs were macroscopically characterized by the presence of a smooth, regular endovascular surface, whereas diseased aortas displayed FSs that resembled thin yellow deposits that were always localized on the dorsal portion of the aorta, facing the intercostal/lumbar artery ostia. In the assessed FA lesions, white capsules were found surrounding the yellow deposits (Figure 1, bottom row). Histologically, the FSs were composed of a large number of cells, whereas the lipid centers of the FAs were acellular and devoid of nuclei (Figure 2A).

Masson’s trichrome staining (Figure 1) showed that the FS and FA samples were characterized by the presence of foam cells, resulting in a translucent or granular cytosolic appearance. Co-staining with Perls + DAB revealed that granular foam cells in the FS and FA samples accumulated redox active iron. Perls alone and DAB alone are strictly negative. In the HA samples, only a few focal iron deposits were observed (Figure 1). 147 sections (each from a different donor) of early-stage human aortic lesions were stained with Perls, DAB, or both (Perls + DAB). The Perls + DAB co-staining revealed that redox-active iron was detected more frequently in the FS (84%) and FA (90%) samples than in the HA (22%) samples (*p* < 0.001, Table 2). Staining with Perls alone, which reveals the ferritin-associated FeIII with a low oxidative potential, was always negative in the tunica intimas of the HA samples and was positive in 9 and 39% of the FS and FA samples, respectively (Table 2). Similarly, staining with DAB alone, which reveals the pseudo-peroxidase activity from intact RBCs, was always negative in the tunica intimas and tunica medias from the HA samples, while 16% of the FS samples and 26% of the FA samples were positive (Table 2). Altogether, these data indicate that the redox-active iron is present as soon as FS and that iron-dependent oxidative stress is an early hallmark of plaque development.

To identify hemoglobin as a possible source of redox-active iron, we immunostained tissue sections with an anti-hemoglobin antibody. Hemoglobin was detected to a more or less extent on all pathological sections. In FS samples, hemoglobin was focally detected in the endothelium and in the vSMC-rich subendothelial intima (Figure 2A, a). In FA samples, the presence of hemoglobin was detected in the lipid core (Figure 2A, b). Frequently, Hb staining co-localized with MYH-11 staining, corresponding to vSMCs. These qualitative results were confirmed by the measure of intimal heme content. We observed a trend for higher heme content in FS samples (Figure 2B). The tissue content of heme was significantly elevated in FA as compared to FS samples (*p* < 0.05) and HA (*p* < 0.01).

### Glycophorin A and Ceroids Can Be Detected in Early Aortic Lesions

**Figure 3:**
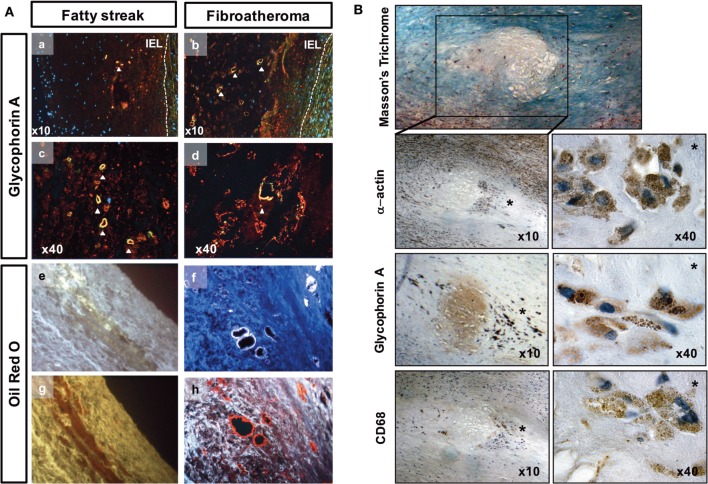
Red blood cell phagocytosis in early-stage atheroma. **(A)** Detection of ceroids (autofluorescence) and glycophorin A (red) in the tunica intima of fatty streaks (FSs) and in lipid core of fibroatheromas (FAs) corresponding to high magnifications of pictures 2Aa (FS), b (FA), respectively. Granular and ring-shaped ceroids (merged yellow) co-localized (a–d) with glycophorin A immunostaining. Autofluorescent granular ceroids (e) and ceroid rings (f) were detected in the tunica intimas of FS and Fas, respectively (white autofluorescence at a wavelength of 350 nm). The ceroids were also characterized by their ability to bind ORO (g,h, red staining). IEL, internal elastic lamina. **(B)** Photographs of a lipid core surrounded by phagocytes in the shoulder region (*) of an initial plaque (Masson’s trichrome). These phagocytes are α-actin^+^, glycophorin A (Gly-A)^+^, and CD68^+^. Gly-A and CD68 specifically localized to cytosolic granules in the phagocytes (40×).

To accurately identify the presence of RBCs, we immunostained histological sections of FS and FA samples with an antibody against glycophorin A (Gly-A), which is a RBC membrane marker (Figure 3A, a–d). Gly-A immunostaining appeared as small granular points that could be differentiated from autofluorescence. Indeed, autofluorescent structures corresponding to ceroids (35) were often observed and co-localize with Gly-A (merged yellow fluorescence). We observed that these autofluorescent ceroids were present at a significantly lower frequency in the HA samples (23%; *p* < 0.001) compared with those of the FS (71%) and FA samples (94%); not significantly different from FS samples (Table 1; Figure 3A, e,f). In addition, the ceroids predominantly appeared as round granules (Figure 3A, e) in the HA and FS samples, whereas ceroid rings were more often observed in the FA samples (Figure 3A, f). As a lipofuscin pigment produced by oxidation, ceroids covalently link lipids to proteins and therefore could also be detected by ORO staining, despite the delipidation of the deparaffinized sections (Figure 3A, g,h).

Importantly, Gly-A was commonly associated with ceroids in the FS and FA samples. Gly-A was also found in the protein background of the lipid core and was associated with α-actin and CD68 staining [Figure 3B, note that CD68 is a marker of phagolysosome activity (36) within phagocytes and can be expressed by vSMCs (37)].

**Figure 4:**
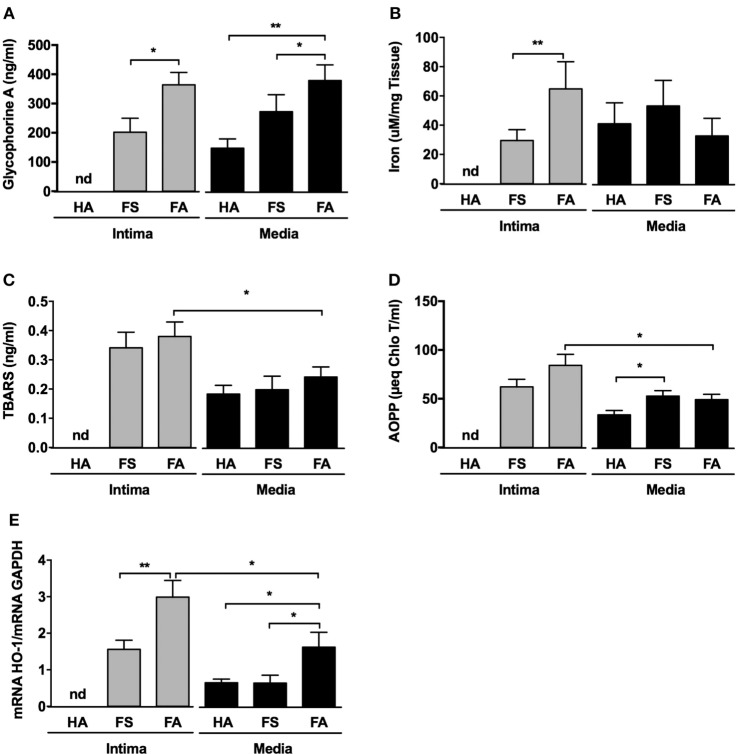
Fatty streaks (FSs) and fibroatheromas (FAs) display high glycophorin A concentration, increased iron content, and augmented oxidative stress. **(A)** Glycophorin A concentration (*n* = 10) was determined in the conditioned media of healthy aortas (HAs), FSs, and FAs. **(B)** Iron content was evaluated in the corresponding tissues. **(C,D)** Thiobarbituric acid reactive substance (TBARS) [*n* = 10, lipids, panel **(C)**] and advanced oxidation protein product (AOPP) [*n* = 10, proteins, panel **(D)**] concentrations were determined in the same conditioned media described above. (E) The expression of Heme-Oxygenase-1 (HO-1) was evaluated in the intima and the tunica medias of healthy aortas (HA, *n* = 10) and from the tunica intimas (*n* = 10) and tunica medias (*n* = 10) of fatty streaks (FSs) and fibroatheromas (FAs). *p < 0.05, **p < 0.01; Mann–Whitney’s test.

We also found that Gly-A concentrations (Figure 4A) were significantly increased in the conditioned medium of the intimas of the FA samples compared with the conditioned medium of the intimas from the FS samples. Notably, for the HA samples, it was virtually impossible to separate and process the tunica intima, as indicated by “nd” in Figure 4 and the following figures. Gly-A concentrations were also significantly increased in the conditioned medium of the intimas from the FS and FA samples compared with those from the HA samples. Similarly, iron concentrations were significantly increased in the tunica intimas of the FA samples compared with those of the FS samples (Figure 4B). These results are consistent with the increased immunodetection of Gly-A in the lesions (Figure 3).

Together, these results indicate that augmented iron deposition and increased quantities of RBC-derived products in the arterial wall characterize both the initiation of FS formation and the progression toward FA and can be further associated with vSMC markers. Additionally, the presence of ceroids, which are insoluble polymers of oxidized lipids and proteins, and their frequent co-localization with Gly-A, indicate that RBC infiltration may promote local oxidative stress.

### Oxidative Stress Is Increased in Early Atheroma

Thiobarbituric acid reactive substance (Figure 4C) concentrations were augmented in the conditioned medium prepared from the tunica intimas compared with that of the matched tunica medias. This difference was only statistically significant for the FA samples, and the concentrations tended to increase in the FS intimas. AOPPs concentrations (Figure 4D) were also significantly increased in the conditioned medium from the tunica intimas of the FA samples compared to media. Increased oxidation status was associated with significant overexpression of heme oxygenase-1 (HO-1) (Figure 4E) in the tunica intimas of the FS and FA samples compared with that of the corresponding tunica medias. The tunica medias of the FA samples had a higher oxidation status than did those of the FS samples.

### Iron Metabolism Pathway Gene Expression Is Altered in Early Human Atheroma

**Figure 5:**
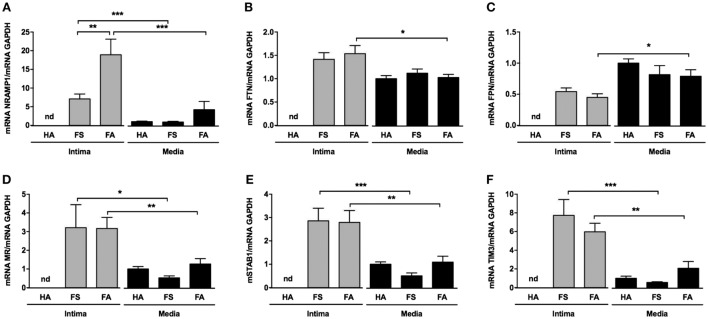
Intraparietal red blood cells are associated with iron storage and expression of scavenger receptors. **(A–C)** mRNA levels of the following iron metabolism genes: NRAMP, ferritin, and ferroportin [**(A–C)**, respectively]. **(D–F)** mRNA levels of the following phagocytosis genes: stabilin-1, mannose receptor (MR), and TIM3 [**(D–F)**, respectively]. These quantifications were performed from mRNA isolated from the tunica medias of healthy aortas (HAs, *n* = 10) and from the tunica intimas (*n* = 10) and tunica medias (*n* = 10) of fatty streaks and fibroatheromas (FAs). *p < 0.05; **p < 0.01; ***p < 0.0001 (Mann–Whitney’s test).

The transcripts for NRAMP1 (a protein involved in iron transport between cell phagolysosomes and cytosol) and ferritin (a protein involved in iron storage) were significantly increased in the intimas of the FA samples (Figures 5A,B, respectively). Conversely, the transcription of ferroportin (a protein involved in iron export from cells) was decreased in the intimas of the FS (ns) and FA (*p* < 0.05) samples compared with that of the corresponding tunica media samples, suggesting iron retention within foam cells (Figure 5C).

In parallel, the levels of various transcripts for receptors potentially involved in phagocytosis, such as TIM-3, stabilin-1 (STAB1) and mannose receptor (MR) (CD206), were significantly increased in the tunica intimas of the FS and FA samples compared with that of their corresponding tunica medias (Figures 5D–F, respectively).

### Senescent RBCs Are Phagocytized by Primary vSMCs *In Vitro*

**Figure 6:**
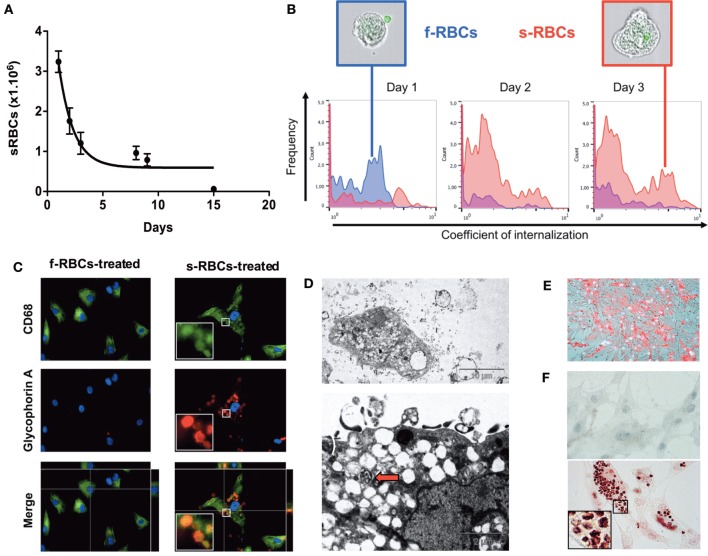
Vascular smooth muscle cells (vSMCs) phagocytose senescent red blood cells (sRBCs) *in vitro*. **(A)** Time course of the diminution of sRBC in the culture medium of vSMCs. The purity of the culture was obtained by dissecting the media form the adventitia. The vSMC nature was validated by α-actin and transgelin staining (not shown) **(B)** Internalization coefficient of RBCs in resuspended vSMCs at days 1, 2, and 3 (*n* = 5 for each time point) calculated by the IDEAS^®^ software (Amnis). vSMCs with a low coefficient are plotted on left side of each histogram, as observed with fresh RBCs (f-RBCs; blue curves; note the presence of a CFSE + RBC outside of the vSMC in the representative image in the blue-colored frame). vSMCs with a high internalization coefficient will be displayed on the right side, as illustrated with vSMCs co-cultured with senescent RBCs (sRBCs; red curves; an internalized CFSE + RBC can be detected inside the vSMC shown in the red-colored frame). **(C)** Glycophorin A and CD68 staining of vSMCs incubated with fresh RBCs or with senescent RBCs for 3 days. **(D)** Electron microscopy of vSMCs that were co-cultured with RBCs. Note that vSMCs internalized sRBCs, formed and released exosomes, retained lipophagic translucent vacuoles that endowed these cells with a foam cell-like phenotype, and displayed phagolysosomes (red arrow). **(E)** Oil Red O staining of membrane cholesterol from RBCs incorporated by vSMCs. **(F)** Perls + DAB staining of redox-active iron in vSMCs incubated with fresh RBCs (higher panel, negative) or with senescent RBCs (lower panel, positive) for 3 days.

We co-incubated either fresh or senescent RBCs (sRBCs), overexposing PS (2% for fresh RBC, 98% for senescent RBC) with human vSMCs in primary cultures. sRBC clearance by vSMCs was evaluated *via* kinetic counting of the remaining RBCs in the culture medium (Figure 6A). At 3 days of incubation, 76% of the sRBC were phagocytosed by vSMC. We also used a quantitative image stream approach (AMNIS), which showed an increased percentage of internalized sRBCs within vSMCs and an augmented coefficient of internalization after one day of incubation compared with the corresponding values obtained using fresh RBCs (Figure 6B).

Phagocytosis of sRBCs by human vSMCs was evaluated by Gly-A staining after 3 days of incubation (Figure 6C). We found that Gly-A signals drastically increased in vSMCs that were incubated with sRBCs compared with those that were incubated with fresh RBCs. Importantly, the Gly-A-positive cells were also positive for CD68.

Electron microscopy was used to confirm that vSMCs not only can engulf sRBCs but form and release exosomes, and retain lipophagic vacuoles, thereby endowing these cells with a foam cell-like phenotype, but also can activate phagolysosomes, which degrade RBC structures (Figure 6D).

### Primary vSMCs Produce ROS after sRBC Phagocytosis

**Figure 7:**
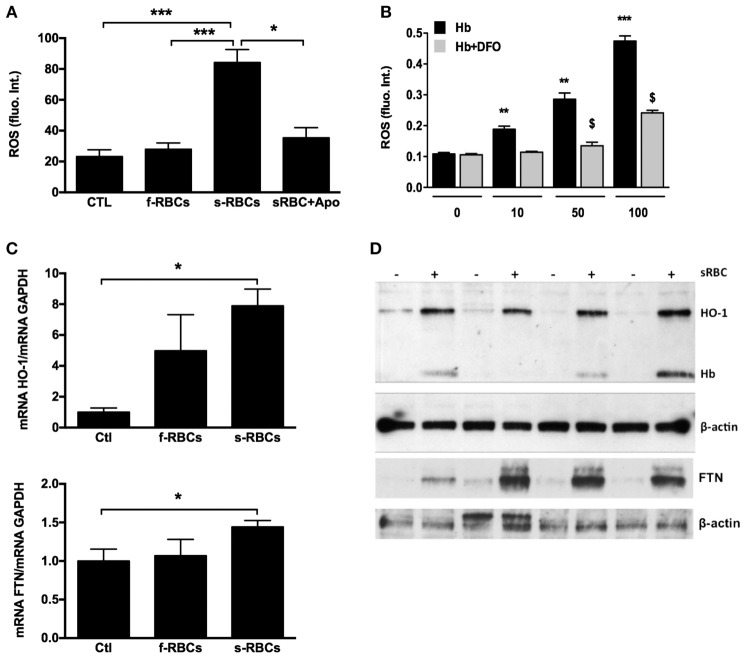
Senescent red blood cell (sRBC) phagocytosis induces reactive oxygen species (ROS) production and heme oxygenase-1 (HO-1) and ferritin expression. **(A)** Vascular smooth muscle cells (vSMCs) were incubated with fresh or RBCs (1 × 10^6^ cells) for 3 days (*n* = 6), and ROS production was assessed by fluorometry. **(B)** vSMCs were incubated with increasing concentrations of hemoglobin (0–100 µM) ± desferoxiamine (20 µM) for 2 h (*n* = 6), and ROS production was assessed as in **(A)**. **(C,D)** HO-1 and ferritin expression in vSMCs after 3 days of incubation with senescent RBCs (*n* = 4) were determined by RT-PCR **(C)** and Western blot **(D)**. Intracellular hemoglobin content **(D)** was determined by Western blot.

We also observed that human cultured vSMCs engulfing sRBCs accumulated lipids and became ORO-positive (Figure 6E). In addition, phagocytosis of sRBCs by vSMCs resulted in oxidative stress, as shown by strong Perls + DAB staining of redox-active iron (Figure 6F) and a significant increase in reactive oxygen species (ROS) production (Figure 7A). The increased ROS production was dependent on NAPDH oxidase activity because the addition of apocynin, a NAPDH-specific inhibitor, strongly decreased ROS production (Figure 7A). We also observed that incubating vSMCs with Hb led to a dose-dependent increase in ROS production, which was partially blocked by desferoxiamine, providing evidence of the catalytic role of redox-active iron on endogenous oxidase activities (Figure 7B). In response, erythrophagy induced HO-1 and ferritin expression in vSMCs at both the mRNA (Figure 7C) and protein levels (Figure 7D). Importantly, Hb and HO-1 were concomitantly detected (Western blot) in vSMCs only when they were co-cultured with sRBCs (Figure 7D).

### Hypercholesterolemia in Rabbits Affects RBC Trafficking in the Aortic Intima

**Figure 8:**
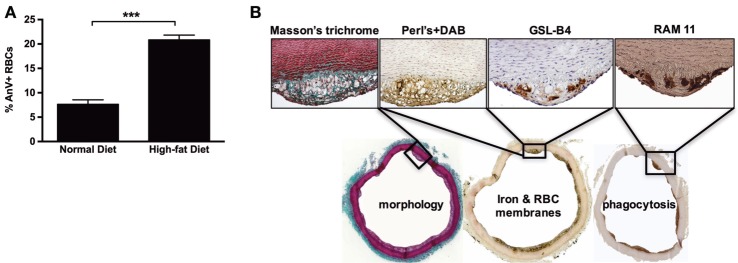
High-cholesterol diet induces red blood cell accumulation in rabbit foam cells. **(A)** After consuming a hypercholesterolemic diet for 3 months, rabbits showed significantly increased percentages of circulating Annexin V^+^ RBCs (***p < 0.001; Mann–Whitney’s test). **(B)** The consumption of a hypercholesterolemic diet was associated with the development of focal subendothelial lesions characterized by the predominance of foam cells, as shown by Masson’s trichrome staining. These foam cells were redox-active iron-positive based on Perls + DiAminoBenzidin (DAB) staining (Perls alone and DAB alone were strictly negative, not shown). They were also recognized by a rabbit RBC membrane marker (GSL-B4 lectin) and were found to express a phagocyte marker (RAM11).

We next tested whether experimental hypercholesterolemia in rabbits could reproduce some of our findings from human samples. As expected, after consuming a cholesterol-rich diet for 6 weeks, the rabbits developed aortic lipid-rich intimal atheroma. We also observed that diet-induced hypercholesterolemia led to a significant increase (×3) in the proportion of circulating Annexin V^+^ RBCs (Figure 8A). Strikingly, in the intimal lesions that developed in these hypercholesterolemic rabbits (Masson’s trichrome), RBCs (GSL-B4+) were closely associated with strong RAM11 immunostaining and presence of redox-active iron (Perls + DAB staining; Figure 8B). These results indicate that FS lesions in hypercholesterolemic rabbits also contain iron deposits and infiltrated RBCs, in agreement with our observations of human FS and FA samples.

### An Overload of Circulating sRBCs Leads to Increased Arterial Iron Content in a Rat Model

**Figure 9:**
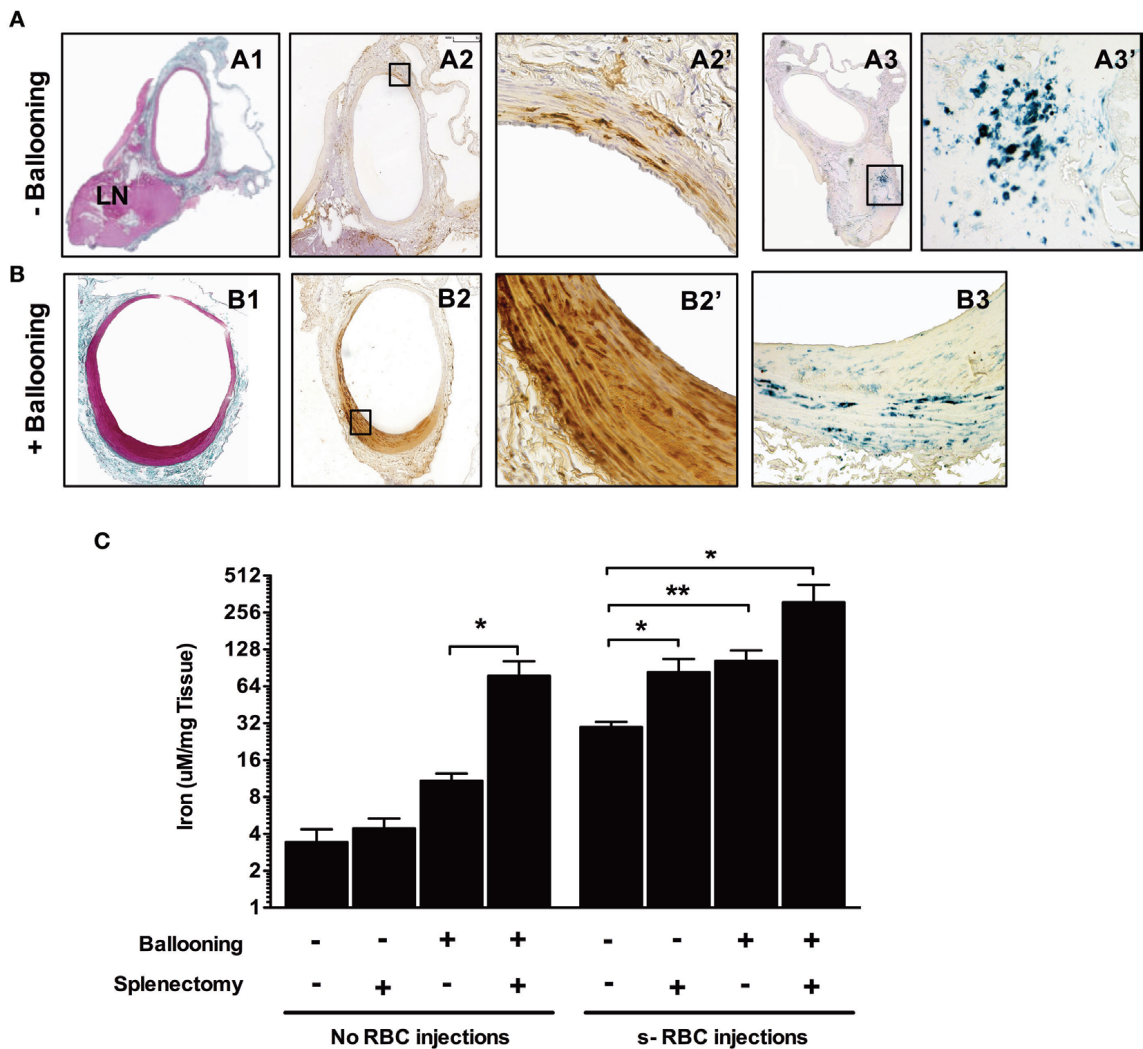
Balloon injury promotes red blood cell phagocytosis. Rats were either subjected to intravenous injections of senescent RBCs once a week for 2 weeks or were not. Subsequently, a subset of the rats was submitted to ballooning of the abdominal aorta. [line **(A)**] Aorta from a rat injected with senescent RBCs without ballooning. Masson’s trichrome staining (A1) of the aorta associated with an adjacent lymph node. Areas positive for Perls + DiAminoBenzidin (DAB) stainig (A2, A2′) were observed throughout the media, indicating the presence of redox-active iron (negative for Perls or DAB alone, not shown). Perls staining (A3, A3′) revealed a continuum of iron transport and metabolism between the arterial wall and the local LN. **(B)** Aorta of a rat injected with senescent RBCs after ballooning. Masson’s trichrome staining (B1) showing intimal proliferation. Perls + DAB staining (B2, B2′) indicating the presence of redox-active iron in the intima and media. Perls alone staining (B3), showing an accumulation of ferric iron (sequestered form potentially linked to ferritin) in the arterial wall. **(C)** Concentrations (logarithmic scale) of iron in the aortas of different groups of rats following senescent RBC injections, ballooning and/or splenectomy (*n* = 10 for each group), as determined by spectrophotometry. *p < 0.05 and **p < 0.01; Mann–Whitney’s test.

To experimentally assess RBC entry into the vascular wall and to determine whether the status of the endothelium is critical for this process to occur, we submitted inbred Lewis rats to repeated injections of isogenic rat sRBCs. Two groups of rats were evaluated: in the first the aorta was left untouched, while the aorta was balloon-injured in the second. To further increase sRBC concentrations, a subset of the rats was splenectomized. The repeated injection of sRBCs significantly increased both average spleen weight (from 249.3 ± 67 mg in the controls to 462.4 ± 140 mg in the experimental group; *p* = 0.0002) and average spleen iron concentration (314 ± 33 versus 469 ± 40 μM/mg tissue; *p* < 0.01). Positive Perls, DAB, and Perls + DAB staining was found in the isolated aortas (Figure 9A). The para-aortic lymph nodes were also positive for Perls (Figure 9A). When sRBC injections were combined with ballooning and/or splenectomy, all of the above parameters significantly increased (Figure 9B). Factorial analysis of variance established that the injection of sRBCs was the most important factor underlying the increased aortic iron content (Figure 9C; *p* < 0.007). However, ballooning and splenectomy also promoted iron accumulation in the aortic wall, even if their effects were more modest (*p* = 0.015 and *p* = 0.036, respectively). There were no significant interactions between the three factors.

## Discussion

Our present observations confirm that FS and early FA are initiated in juxta intercostal/lumbar artery ostia in the dorsal part of the human aorta (4). Early FSs evolve into FAs, in which, intimal lipid deposits are recovered by the fibrocellular proliferation of vSMCs, while the lipid core of the initial plaque becomes acellular. Foam cells, characterized by intracellular accumulation of lipids, are observed during these early stages. Our present observations also confirm that intimal vSMCs have been pointed as key funders of foam cells and phagocytosis in the arterial wall (9).

In this context of human early atheroma, the roles of RBCs have not yet been fully recognized. Early studies focused mainly on the role of free hemoglobin on endothelial cells (18, 38) and of RBC in the late stages (21, 22) of atherothrombotic evolution. In the present study, we explored the possibility that RBC interactions with the wall and sRBC efferocytosis by vSMCs could be early priming events underlying lesion development in human arteries. Toward this end, we co-stained cells with Perls + DAB (34) to enhance detection redox-active iron, whereas Perls alone and DAB alone served as negative controls. The results provided evidence that this redox-active iron can catalyze endogenous oxidase activities, as shown by the DAB polymerization (32). Although no selection of tissue samples was performed, and despite the limited sample size, we nonetheless found a high percentage of tissue sections with FS or FA lesions in which redox-active iron was detected using this simple histochemical method. Redox-active iron was mainly detected in foam cells regardless of lesion stage. The presence of iron in these cells led them to adopt a granular, gray appearance, while simple lipid deposits appeared as translucent objects, as previously described by electron microscopy (39). Of note, intimal redox-active iron could be detected in apparently HAs, suggesting that iron acquisition by the intima, is a common occurrence and may represent an early event in the development of atheroma. In contrast, when evaluating ferric iron storage by staining with Perls alone, significantly less iron accumulation was found, demonstrating the low sensitivity of Perls staining for detecting redox active iron.

These data were confirmed by hemoglobin and heme detection in human FS and FA samples. Immunohistochemistry of hemoglobin (detecting the protein part of Hb) showed that hemoglobin is detected at a high frequency in FS and FA samples. Importantly, hemoglobin detection could be associated with the detection of vSMC myosin, suggesting that the vSMCs could be the effector and the target of intracellular Hb release. Heme assay showed an increase of the tissue heme content, which followed the progression of the pathology. In this context, Hb and heme appear as molecular intermediate metabolites between sRBC efferocytosis and intracellular iron accumulation.

Strikingly, the frequency of redox-active iron staining was similar to the frequency with which we detected ceroid lipofuscin. Ceroids are autofluorescent, insoluble polymers of oxidized lipids and proteins. They can form intracellularly (40), appearing as punctate, small particles of gruel, depending on the presence of redox-active iron and phagolysosome activity (41), or they can form extracellularly, appearing as rings. Our observations that ceroids co-localized with redox-active iron deposits and myosin within the cell and further in the extracellular compartment (42, 43) might indicate that ceroids primarily could form within the intracellular compartment of vSMC. Importantly, results from Raman and fluorescence spectroscopy have been recently used to demonstrate the presence of Hb and oxidase within ceroids (44).

To determine whether intimal redox-active iron is derived from RBCs or from free Hb, we stained tissue sections with an anti-Gly-A antibody to visualize the presence of RBC membranes (21). In the FS and FA samples, iron and Gly-A were often co-localized. RBC membranes provide an important source of unesterified cholesterol in advanced hemorrhagic plaques (21). The presence of Gly-A in the FS and FA samples strongly suggests that cholesterol derived from RBC membranes is also a significant contributor of engulfed and extracellular cholesterol in early lesions, as has shown for advanced lesions (20, 21). Moreover, in atheromatous tissues, we observed that intracellular Gly-A could co-localize with CD68^+^ phagolysosomal structures and with intracellular or extracellular ceroids in foam cells and lipid core. We also observed the presence of glycophorin A in ceroids providing evidence that one part of the oxidized lipids came from RBC membranes in ceroids. The co-localization of redox-active iron, lipids, and Gly-A and the involvement of the phagolysosome in ceroid formation suggest that these pathological structures are closely associated with senescent erythrocyte efferocytosis in early atheroma.

We also observed greater release of TBARS and AOPPs, markers of lipid and protein oxidation, respectively, from the intima of early lesions than from the corresponding tunica medias, suggesting that the oxidation process initiates in the intima. In direct response to heme, activation of NF-KB (38), induction of ferritin (45), and heme-oxygenase-1 (HO-1) (46) partially protect endothelial cells from oxidative injury. We detected HO-1 overexpression in our FS and FA samples, which could act as a compensatory mechanism, explaining the limited increases in AOPPs and TBARS concentrations observed in cell-rich FSs. This compensatory mechanism would be more limited in the lipid core because of the loss of vSMCs. In parallel, the expression levels of mRNA encoding proteins involved in intracellular iron metabolism were also modified. In particular, NRAMP1, which acts to transport divalent iron across the phagolysosomal membrane (47), and ferritin (48), an ubiquitous intracellular protein that stores and releases iron (49), were upregulated. Conversely, ferroportin-1, a transmembrane iron exporter (50), was downregulated. Taken together, these observations suggest that iron storage is increased in the atherosclerotic vessel wall. Moreover, several proteins that promote phagocytosis after the recognition of exposed PS were also upregulated in FS and FA samples, including TIM3, which possesses a domain allowing for highly specific recognition of exposed PS (51); STAB1, a scavenger receptor that interacts with numerous ligands, including PS (52); and MR, also known as CD206, a C-type lectin primarily present on the surfaces of phagocytes that can recognize various cell-surface saccharide structures (53). Together, the above data strongly suggest that the trafficking of RBCs across the intima is associated with increased iron storage and an increased capacity to recognize and uptake senescent cells. These events could favor the formation of foam cells and the release of redox-active iron from RBC/heme, which could be instrumental in inducing intimal oxidative processes.

To directly test this hypothesis, we incubated senescent or fresh RBCs with primary cultures of vSMCs. vSMCs were chosen because they are the main resident stromal cells of the arterial wall and are predominantly involved in FSs and in the fibrocellular caps of FAs. vSMCs are highly plastic (13, 54); they can acquire a CD68^+^ macrophage-like phenotype (8, 10–12) and can phagocytose sRBCs (23). The transient presence of Gly-A and hemoglobin, the retention of redox-active iron, the formation of lipid vacuoles, and the intense activation of the phagolysosome and exocytosis provide evidences of erythrophagy by vSMCs. As in tissues, sRBC efferocytose by vSMCs promoted oxidation by catalyzing oxidase activity and induced HO-1 and ferritin expression as factors limiting oxidative injury (45, 46).

Finally, we explored the relevance of the above mechanism *in vivo* in experimental models. The induction of atheroma in rabbits *via* the provision of a cholesterol-rich diet is a well-established model that can be characterized by the formation of a proliferative neo-intima that is particularly rich in lipid-laden foam cells and originates, at least in part, from resident vSMCs (55). In an interesting study, Nakayama and colleagues explored HO-1 and bilirubin expression in a high-fat-diet rabbit model (56). They found that intimal foam cells express and accumulate HO-1 and bilirubin (a product of heme degradation), suggesting that these cells metabolized heme. However, the above studies did not address the origin of the heme. In parallel, Pang and colleagues reported ferritin overexpression in the aortas of hypercholesterolemic rabbits (48), and Lee et al. reported the presence of iron (based on Perls + DAB histochemistry and electron microscopy results) in rabbit foam cells (57). In the present study, we identified RBCs as a potential source of heme because the intimal foam cells in our hypercholesterolemic rabbits contained redox-active iron, showed phagolysosome activation (RAM-11) and stained positive for RBC cell-surface saccharides (isolectin B4). Although we recognize the poor specificity of this marker, to the best of our knowledge, it is the only RBC membrane marker available for use in rabbits.

Hypercholesterolemia is known to impact RBC membrane function by chelating chemokines through the Duffy antigen, promoting erythrophagy through PS exposure, and augmenting interactions between endothelial cells and leukocytes in rabbits (30), mice (58), and humans (59). Accordingly, we found an increased percentage of sRBCs in our hypercholesterolemic rabbits. Therefore, by enhancing the proportion of sRBCs, a hypercholesterolemic status can potentially increase RBC interactions with the arterial wall.

Therefore, we next tested whether RBCs must be senescent to interact with cells in the vascular wall and whether the vascular endothelium must be intact to support these interactions. For this purpose, we developed a model of repeated intravenous injections of sRBCs in rats. Aortic endothelium was either left untouched or mechanically removed. In addition, a subset of the rats was subjected to splenectomy. Splenectomy was expected to significantly reduce the clearance of circulating sRBCs. It is important to note that rats are particularly resistant to atheroma. Strikingly, we observed that intravenous injection of sRBCs led to their convection across the arterial wall and to iron retention within the aorta. This iron retention was significantly enhanced by ballooning and/or splenectomy. These data strongly suggest that RBC trafficking across the aortic wall could be a pathogenic event that increases the iron content within the wall. The mechanism by which RBCs can traffic across the vessel wall remains to be clarified. Our data indicate that the integrity of the endothelium offers only relative protection to the arterial wall. While we have shown that erythrophagy by vSMCs can indeed lead to the transformation of vSMC into foam cells and increase their oxidative potential. We recognize that other cells might display the same capacities. In addition, our study does not exclude the possibility that the release and endocytosis of free Hb can occur independently of erythrophagy. Nevertheless, we washed our senescent RBCs in order to focus on RBC rather than to free hemoglobin. Results of our experimental design in rats are similar to the results published by Baek et al. (19), showing that transfusion of long-term stored RBCs (likely similar to the sRBCs that we used in our experiments), sensitive to spontaneous hemolysis, was highly toxic for the arterial wall in guinea-pigs. In order to minimize the direct toxic effect of Hb, fresh and sRBC were washed before use in our study.

The clinical significance of our findings is multifaceted. Indeed, the major risk factors (i.e., hypercholesterolemia, smoking, diabetes, aging) can alter the biology of RBCs. We have observed that hypercholesterolemia induced a three-fold increase in the proportion of senescent RBCs in rabbits. This has been also reported from the use of a high-fat-diet mouse model (58). Diabetes as well as smoking (60) was also reported to increase the proportion of sRBCs in human. Therefore, our study indicates the need for a thorough evaluation of RBC status in atherothrombosis clinical studies to test whether risk factors with unknown pathophysiological mechanisms could act through an effect on RBCs, to evaluate whether current drugs act on RBCs, and to design new treatments aimed at preventing RBC dysfunction.

Most atherothrombosis studies focus on leukocytes. This study is unique because it focused on erythrocytes. In evaluating human tissues, cell cultures, and experimental animal models, we have demonstrated that RBCs can enter the arterial wall during the earliest stages of atheroma and are phagocytosed by vSMC. This entry serves as a pathogenic mechanism and is enhanced by hypercholesterolemia, endothelial injury, and RBC senescence. Because RBCs carry cholesterol and phospholipids in their membranes and are an iron/heme cargo, their infiltration and phagocytosis by vSMCs appear to be instrumental processes underlying the formation of foam cells, the genesis of intraparietal oxidation and finally the anti-oxidant response of vSMC. Therefore, this study unravels a previously unforeseen pathophysiological mechanism of plaque formation where erythrocyte angiophagy by vSMCs is a key developmental trigger.

## Ethics Statement

Human aortas were consecutively collected from deceased organ donors from 2010 to 2013 under the authorization of the French Biomedicine Agency (PFS 09-007). All experimental and animal care procedures complied with the principles formulated by the National Society for Medical Research (animal facility agreement: No. B75-18-03). This study was performed under the advice of our institution’s animal ethical committee (authorization no. 75-101) and complied with all guidelines for animal experimentation set forth by the French Ministry of Agriculture.

## Author Contributions

Study design: SD, RB, KG, AN, and J-BM. Acquisition of the data: SD, RB, BH-T-N, JL, ZT, VO, CD, MM, LL, and KG. Analysis and interpretation of the data: SD, RB, BH-T-N, AN, and J-BM. Manuscript preparation: SD, BH-T-N, KG, AN, and J-BM.

## Conflict of Interest Statement

The authors declare that the research was conducted in the absence of any commercial or financial relationships that could be construed as a potential conflict of interest.
